# Emergency care in 59 low- and middle-income countries: a systematic review

**DOI:** 10.2471/BLT.14.148338

**Published:** 2015-05-26

**Authors:** Ziad Obermeyer, Samer Abujaber, Maggie Makar, Samantha Stoll, Stephanie R Kayden, Lee A Wallis, Teri A Reynolds, on behalf of the Acute Care Development Consortium

**Affiliations:** a Department of Emergency Medicine, Harvard Medical School, 75 Francis Street, Boston, MA 02115, United States of America (USA).; b Brigham and Women’s Hospital, Boston, USA.; c Harvard Affiliated Emergency Medicine Residency Program, Boston, USA.; d University of Cape Town, Cape Town, South Africa.; e University of California at San Francisco, San Francisco, USA.

## Abstract

**Objective:**

To conduct a systematic review of emergency care in low- and middle-income countries (LMICs).

**Methods:**

We searched PubMed, CINAHL and World Health Organization (WHO) databases for reports describing facility-based emergency care and obtained unpublished data from a network of clinicians and researchers. We screened articles for inclusion based on their titles and abstracts in English or French. We extracted data on patient outcomes and demographics as well as facility and provider characteristics. Analyses were restricted to reports published from 1990 onwards.

**Findings:**

We identified 195 reports concerning 192 facilities in 59 countries. Most were academically-affiliated hospitals in urban areas. The median mortality within emergency departments was 1.8% (interquartile range, IQR: 0.2–5.1%). Mortality was relatively high in paediatric facilities (median: 4.8%; IQR: 2.3–8.4%) and in sub-Saharan Africa (median: 3.4%; IQR: 0.5–6.3%). The median number of patients was 30 000 per year (IQR: 10 296–60 000), most of whom were young (median age: 35 years; IQR: 6.9–41.0) and male (median: 55.7%; IQR: 50.0–59.2%). Most facilities were staffed either by physicians-in-training or by physicians whose level of training was unspecified. Very few of these providers had specialist training in emergency care.

**Conclusion:**

Available data on emergency care in LMICs indicate high patient loads and mortality, particularly in sub-Saharan Africa, where a substantial proportion of all deaths may occur in emergency departments. The combination of high volume and the urgency of treatment make emergency care an important area of focus for interventions aimed at reducing mortality in these settings.

## Résumé

**Objectif:**

Réaliser un examen systématique des soins d'urgence dans les pays à revenu faible ou intermédiaire (PRFI).

**Méthodes:**

Nous avons recherché dans les bases de données de PubMed, CINAHL et de l'Organisation mondiale de la Santé des rapports décrivant les soins d'urgence dispensés dans les établissements médicaux et obtenu des données non publiées auprès d'un réseau de cliniciens et de chercheurs. Nous avons sélectionné plusieurs articles à inclure d'après leur titre et leur résumé en anglais ou en français. Nous avons extrait des données liées à l'état de santé des patients, à la démographie et aux caractéristiques des établissements et des prestataires. Les analyses se sont limitées à des rapports publiés à partir de 1990.

**Résultats:**

Nous avons identifié 195 rapports relatifs à 192 établissements implantés dans 59 pays. Il s'agissait pour la plupart d'hôpitaux universitaires situés dans des zones urbaines. La mortalité moyenne au sein des services d'urgence était de 1,8% (intervalle interquartile, IQR: 0,2–5,1%). Elle était relativement élevée dans les centres pédiatriques (moyenne: 4,8%; IQR: 2,3–8,4%) et en Afrique subsaharienne (moyenne: 3,4%; IQR: 0,5–6,3%). Le nombre moyen de patients était de 30 000 par an (IQR: 10 296–60 000), la plupart d'entre eux étant des jeunes (âge médian: 35 ans; IQR: 6,9–41,0) et de sexe masculin (moyenne: 55,7%; IQR: 50,0–59,2%). La majorité des établissements employaient des médecins en formation ou dont le niveau de formation n'était pas précisé. Rares étaient les prestataires à avoir reçu une formation spécialisée en soins d'urgence.

**Conclusion:**

Les données existantes concernant les soins d'urgence dispensés dans les PRFI indiquent un nombre de patients et une mortalité élevés, en particulier en Afrique subsaharienne où une fraction importante de l'ensemble des décès est susceptible de survenir dans les services d'urgence. Compte tenu du nombre élevé et de l'urgence des interventions, les soins d'urgence constituent un domaine d'intérêt important pour les actions visant à réduire la mortalité dans ces lieux.

## Resumen

**Objetivo:**

Realizar una revisión sistemática de la atención de emergencia en países de ingresos medios y bajos (PIMB).

**Métodos:**

Se realizaron búsquedas en las bases de datos de PubMed, CINAHL y la Organización Mundial de la Salud (OMS) para encontrar informes que describieran la atención de emergencia en centros sanitarios y se obtuvieron datos sin publicar de una red de clínicos e investigadores. Se seleccionaron artículos para ser incluidos en base a los títulos y resúmenes en inglés o francés. Se recogieron datos de los resultados y demografías de los pacientes, así como características de las instalaciones y los proveedores. Los análisis se redujeron a informes publicados a partir de 1990.

**Resultados:**

Se identificaron 195 informes referentes a 192 instalaciones en 59 países. La mayoría eran hospitales académicamente afiliados en zonas urbanas. La mortalidad media en los servicios de urgencias era de 1,8% (rango intercuartílico, RIC: 0,2–5,1%). La mortalidad era relativamente alta en los centros pediátricos (mediana: 4,8%; RIC: 2,3–8,4%) y en el África subsahariana (mediana: 3,4%; RIC: 0,5–6,3%). La mediana de pacientes era de 30.000 al año (RIC: 10.296–60.000), la mayoría de los cuales eran jóvenes (mediana de edad: 35 años; RIC: 6,9–41,0) y hombres (mediana: 55,7%; RIC: 50,0–59,2%). El personal de la mayoría de los centros eran médicos en formación o médicos cuyo nivel de formación era indeterminado. Muy pocos de estos proveedores tenían una formación especializada en atención de emergencia.

**Conclusión:**

Los datos disponibles en atención de emergencia en PIMB indican una gran carga de pacientes y mortalidad, en especial en el África subsahariana, donde una proporción sustancial de todas las muertes pueden ocurrir en los servicios de urgencias. La combinación entre una gran carga y la urgencia del tratamiento hacen de la atención de emergencia un área importante en la que centrarse de cara a intervenciones dirigidas a reducir la mortalidad en estos escenarios.

## ملخص

**الغرض:**

إجراء مراجعة منهجية للرعاية المقدمة في حالات الطوارئ في البلدان منخفضة الدخل والبلدان متوسطة الدخل (LMIC).

**الطريقة:**

لقد بحثنا في قواعد البيانات في موقع PubMed، والدليل التراكمي للنشريات في مجال التمريض والمهن الصحية المساعدة (CINAHL)، ومنظمة الصحة العالمية (WHO) عن تقارير تبين الرعاية المقدمة في حالات الطوارئ داخل المؤسسات الصحية. وقد استطلعنا بعض المقالات لتضمينها في المراجعة حسب العناوين والملخصات الواردة باللغة الإنجليزية أو الفرنسية. واستخلصنا بيانات بشأن النتائج المتعلقة بالمرضى والخصائص الديموغرافية وخصائص المؤسسات والجهات المقدمة للخدمات. واقتصرت التحليلات على التقارير المنشورة منذ عام **  **1990.

**النتائج:**

توصلنا إلى تحديد 195 **  **تقريرًا يتعلق بـ **  **192 **  **مؤسسة في 59 **  **دولة. وكان معظم المؤسسات يمثل مستشفيات تابعة لجهات أكاديمية في مناطق حضرية. وبلغ متوسط نسبة الوفيات داخل أقسام الطوارئ **  **1.8% (المدى الربيعي: 0.2–5.1%). وكانت نسبة الوفيات مرتفعة نسبيًا في مؤسسات علاج الأطفال (المتوسط: 4.8%؛ **  **المدى الربيعي: 2.3 **  **– **  **8.4%) وفي الدول الواقعة في جنوب الصحراء الأفريقية (المتوسط: 3.4%؛ المدى الربيعي: 0.5–6.3%). وبلغ متوسط عدد المرضى 30,000 في السنة (المدى الربيعي: 10,296 **  **– 60,000)، حيث كان معظمهم من الشباب (متوسط العمر: 35 عامًا؛ المدى الربيعي: 9.6 **  **– **  **41.0) والذكور (المتوسط: 55.7%؛ المدى الربيعي: 50.0–59.2%). وضم فريق العمل بمعظم المؤسسات إما أطباء في مرحلة التدريب أو أطباء ذوي مستوى غير محدد من التدريب. وتوفر لدى عدد قليل جدًا من هذه الجهات المقدمة للخدمة الصحية تدريب تخصصي في تقديم الرعاية الصحية في حالات الطوارئ.

**الاستنتاج:**

تشير البيانات المتوفرة عن تقديم الرعاية الصحية في حالات الطوارئ في البلدان منخفضة الدخل والبلدان متوسطة الدخل إلى ارتفاع عدد المرضى لدى الأطباء المتدربين وارتفاع عدد الوفيات، خاصةً في الدول الواقعة جنوب الصحراء الأفريقية، حيث يُحتمل وقوع نسبة كبيرة من جميع حالات الوفاة في أقسام الطوارئ. وإن اجتماع عنصر ارتفاع العدد وعنصر الحاجة الملحة للعلاج يجعل تقديم الرعاية الصحية في حالات الطوارئ نقطة هامة تستحق التركيز عليها في التدخلات التي تهدف إلى تقليل نسبة الوفيات في مثل هذه المناطق.

## 摘要

**目的:**

旨在对中低收入国家 (LMIC) 中的急救护理进行系统评审。

**方法:**

我们从 PubMed、CINAHL 和世界卫生组织 (WHO) 数据库中搜索了描述机构中急救护理的报告，并且从临床医生和研究人员网络中获取了未经发布的数据。 我们根据其英语或法语标题和摘要筛选了所包含的文章。 我们针对患者疗效和人口统计以及机构和提供方的特性提取了数据。 分析仅限于自 1990 年以来发布的报告。

**结果:**

我们确定了涉及 59 个国家中 192 家机构的 195 份报告。 其中大部分为城市地区的学院附属医院。 急诊部内的平均死亡率为 1.8%（四分位差，IQR： 0.2 至 5.1%）。死亡率在儿科也相对较高（平均值： 4.8%；IQR： 2.3 至 8.4%），而且在撒哈拉以南的非洲死亡率也比较高（平均值： 3.4%；IQR： 0.5 至 6.3%）。患者平均为每年 30 000 例（IQR： 10 296 至 60 000），其中大部分为年轻人（平均年龄： 35 岁；IQR： 6.9 至 41.0）和男性（平均值： 55.7%；IQR： 50.0 至 59.2%）。大多数机构中的工作人员为实习医生或未指定培训水平的医生。 这些提供方中仅有非常少的一部分接受过急救护理方面的专业培训。

**结论:**

中低收入国家 (LMIC) 中现有的急救护理数据表明患者数量和死亡率很高，特别是在撒哈拉以南的非洲，所有死亡中有相当大的一部分比例是发生在急诊部。 治疗量大并且具有紧急性这两个特点的结合让急救护理在我们采取以降低这些环境中的死亡率为目的的干预措施时成为关注的重点领域。

## Резюме

**Цель:**

Систематизированный анализ неотложной помощи в странах с низким и средним уровнем доходов населения.

**Методы:**

В базах данных PubMed, CINAHL и Всемирной организации здравоохранения (ВОЗ) был проведен поиск отчетов по оказанию неотложной помощи в медицинских учреждениях. Кроме того, были получены неопубликованные данные от сети практикующих врачей и исследователей. Статьи отбирались по названию и аннотации на английском или французском языке. Отбирались данные по результатам лечения пациентов и демографические данные, а также информация о характеристиках медицинского учреждения и поставщиков медицинских услуг. Анализировались только отчеты, опубликованные с 1990 года.

**Результаты:**

Было отобрано 195 отчетов по 192 медицинским учреждениям в 59 странах. Большинство из этих учреждений являются академическими больницами, расположенными в городских районах. Средняя смертность в отделениях неотложной помощи составляла 1,8% (межквартильный размах, МКР: 0,2–5,1%). Смертность была относительно высокой в педиатрических медицинских учреждениях (медиана: 4,8%; МКР: 2,3–8,4%) и в Африке к югу от Сахары (медиана: 3,4%; МКР: 0,5–6,3%). Среднее количество пациентов составляло 30 000 человек в год (МКР: 10 296–60 000), большинство из которых были молодого возраста (средний возраст: 35 лет; МКР: 6,9–41,0) и мужского пола (медиана: 55,7%; МКР: 50,0–59,2%). Большинство медицинских учреждений были укомплектованы врачами-стажерами или врачами, уровень подготовки которых не указывался. Лишь небольшое число из рассмотренных поставщиков медицинских услуг обладали специальной подготовкой в области неотложной помощи.

**Вывод:**

Доступные данные по оказанию неотложной помощи в странах с низким и средним уровнем доходов свидетельствуют о большой численности поступающих больных и высоком уровне смертности, особенно в Африке к югу от Сахары, где существенная доля всех смертельных случаев приходится на отделения неотложной помощи. Высокий объем инцидентов и срочность необходимого лечения свидетельствуют о том, что неотложная помощь является одной из важнейших областей, на которую следует направить мероприятия по снижению уровня смертности в указанных условиях.

## Introduction

Ebola virus disease,^1^ cholera,^2^ armed conflict^3^ and natural disasters^4^ have recently strained systems for the provision of emergency care in low- and middle-income countries (LMICs). Expert groups have voiced concern about these systems’ critical lack of surge capacity and resilience.^5^ Even in non-crisis situations, small surveys^6^^,^^7^ and anecdotal accounts^8^ hint at high volumes of critically-ill patients seeking emergency care in LMICs. This makes emergency care different from other health settings – including primary care – where doctors typically see only 8–10 ambulatory patients per day.^9^

In high-income countries, decades of advances in clinical science and care delivery have dramatically improved process efficiency and patient outcomes for a range of acute conditions.^10^^–^^16^ Despite increasingly urgent calls to apply lessons learnt in high-income countries to LMICs,^17^^–^^19^ a lack of data from the field has made it difficult to convince policy-makers to make major new investments in emergency care. Measuring the state of emergency care in LMICs is challenging, because care is delivered through a heterogeneous network of facilities and medical records are often incomplete, even for basic information such as patient identity and diagnosis.^19^^–^^21^

Because of these challenges, studies of emergency care in LMICs have been limited to small, ad hoc efforts, in individual facilities, that were focused on individual acute diseases and conditions.^22^^–^^28^ We systematically reviewed all available evidence on emergency care delivery to guide future research on – and improvements of – emergency health systems in LMICs.

## Methods

### Systematic search

We did a systematic review (PROSPERO: CRD42014007617) – following PRISMA guidelines^29^ – to identify quantitative data on the delivery of emergency care to an undifferentiated patient population in all LMICs categorized as such in 2013.^30^ To increase capture, we also included the names of the autonomous or semi-autonomous geographical areas recognized by the World Bank^30^ and then disaggregated any relevant data obtained for such areas. For each country or subregion, we searched PubMed, CINAHL and World Health Organization (WHO) regional indices,^31^ using “emerg*” plus the country or area name as the search term. We wished to identify studies of emergency care, irrespective of location, patient complaint or provider specialty. We performed similar searches in Google Scholar but only searched within article titles. We also identified non-indexed journals that regularly published manuscripts on emergency care (available from the corresponding author) and screened every article in every issue of these journals manually. Searches were conducted between 12 August 2013 and 30 May 2014.

We screened reports based on their titles and abstracts in English or French. The full-text potentially relevant articles were retrieved, irrespective of language or date of publication. Since the purpose of our review was to synthesize recent evidence on emergency care, the findings summarized below relate only to data published after 1989. A summary of our observations on data that were published before 1990 is available from the corresponding author. We retained studies describing the delivery of any emergency care in a health facility to adult or paediatric patients, irrespective of the presenting complaint or condition. For each retained article, we conducted backward and forward reference searches: we screened the references cited and, using Google Scholar, we also identified and screened publications that cited the article. We excluded studies that focused on specific conditions or subsets of emergency patients unless they also provided data on the overall population or facility. We also excluded studies that aggregated data from multiple facilities and general descriptions of the state of emergency care in a country. Despite the assistance of trained medical librarians, the full texts of some potentially relevant manuscripts could not be traced. In these cases, we used data from related abstracts or posters, when available.

### Unpublished data

We presented the study protocol and early results at the 2013 African Federation for Emergency Medicine consensus conference. We made use of this presentation and our professional networks to request relevant unpublished data from clinicians in LMICs. Some clinicians, researchers and authors were not authorized to release data that allowed the study health facility or facilities to be identified. In these cases, we identified facilities only by their locations and ownership – i.e. academic, non-profit or for-profit.

### Data extraction

We extracted data on the characteristics of each study facility: country, urban or rural setting, bed count, annual patient volume, ownership and highest level of provider training. We considered a provider to be an emergency physician if reference was made to specialty postgraduate training, board certification or practice within an independent department of emergency medicine. We recorded details of the study population – i.e. age, sex, number of subjects included in analysis, number who arrived by ambulance – the sampling method and key patient outcomes. The latter included the inpatient admission and mortality within the emergency department, the percentages of patients recorded as brought in dead, or dead on arrival, and the length of time each patient stayed in the emergency department.

We created a database containing aggregated study data. When multiple publications described a single facility, we merged them to create a single record that, for each variable of interest, contained the most recently published data available. We stratified facilities using World Bank regions^30^ and considered separately those facilities that only served paediatric populations. If data from a single facility were available disaggregated by age group, we summarized quantitative metrics for adult and paediatric patients separately. Full lists of the included studies and the data extracted and a full description of the study protocol are available from the corresponding author.

### Descriptive analysis

We calculated summary statistics for all relevant metrics that were reported consistently across studies: bed count, annual patient volume, admission and mortality within the emergency department. We made an a priori decision not to perform a formal meta-analysis. Instead, our systematic analysis was meant to capture the distribution of metrics across populations – e.g. adult versus paediatric – and World Bank regions – e.g. Africa versus Asia – as well as global patterns. We thus present means – or medians with interquartile ranges (IQR) – disaggregated by country or region, as appropriate. Statistical analyses were performed using Stata/MP (StataCorp. LP, College Station, United States of America).

## Results

**Fig. 1:**
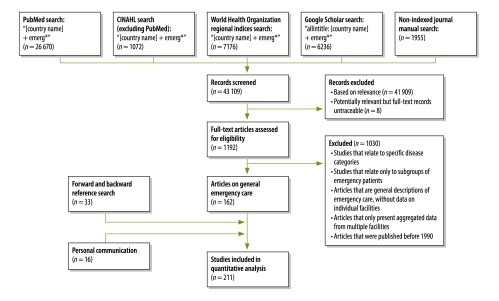
Flowchart for the selection of records on the delivery of emergency care in low- and middle-income countries

Fig. 1 shows the results of our literature search. Of the 195 relevant published studies identified (Table 1; available at: http://www.who.int/bulletin/volumes/93/14/07-148338), 170 (87%) were descriptive reports on hospital-based emergency departments whereas the other 25 (13%) described the impact of an intervention. We obtained relevant unpublished data on a further 16 facilities. After combining multiple reports from the same facility and separating paediatric and adult data – for the three facilities with disaggregated data – we had data on 192 individual facilities in 59 countries. Of the 192 facilities, 107 (56%) were academically affiliated, 11 (6%) were in rural areas and 36 (19%) served paediatric patients exclusively; in the remaining 38, facility type could not be identified. Further information on the health facilities is available from the corresponding author.

**Table 1:** Identified studies on the delivery of emergency care in low- and middle-income countries

Author	Year	Title	Journal	Country or area
A-Rahman NHA	2014	The state of emergency care in the Republic of the Sudan	Afr J Emerg Med	Sudan
Abbadi S *et al*	1997	Emergency medicine in Jordan	Ann Emerg Med	Jordan
Abd Elaal SAM *et al*	2006	The waiting time at emergency departments at Khartoum State-2005	Sudan J Public Health	Sudan
Abdallat AM *et al*	2007	Frequent attenders to the emergency room at Prince Rashed Bin Al-Hassan Hospital	J R Med Serv	Jordan
Abdallat AM *et al*	2000	Who uses the emergency room services?	Eastern Mediterr Health J	Jordan
Abhulimhen-Iyoha BI *et al*	2012	Morbidity and mortality of childhood illnesses at the emergency paediatric unit of the University of Benin Teaching Hospital, Benin City	Nigeria J Pediatr	Nigeria
Adeboye MAN *et al*	2010	Mortality pattern within twenty-four hours of emergency paediatric admission in a resource-poor nation health facility	West Afr J Med	Nigeria
Adesunkanmi ARK *et al*	2002	A five year analysis of death in accident and emergency room of a semi-urban hospital	West Afr J Med	Nigeria
Afuwape OO *et al*	2009	An audit of deaths in the emergency department in the University College Hospital Ibadan	Nigeria J Clin Pract	Nigeria
Aggarwal P *et al*	1995	Utility of an observation unit in the emergency department of a tertiary care hospital in India	European J Emerg Med	India
Akpa MR *et al*	2013	Profile and outcome of medical emergencies in a tertiary health institution in Port Harcourt, Nigeria	Nigeria Health J	Nigeria
Al-Hakimi ASA *et al*	2004	Load and pattern of patients visiting general emergency Al-Thawra Hospital, San a'a	Yemen Health Medical Res J	Yemen
Alagappan K *et al*	1998	Early development of emergency medicine in Chennai (Madras), India	Ann Emerg Med	India
Asumanu E *et al*	2009	Improving emergency attendance and mortality – the case for unit separation	West Afr J Med	Ghana
Atanda HL *et al*	1994	Place des urgences medicales pediatriques dans un service medical a Pointe-Noire	Med Afr Noire	Congo
Avanzi MP *et al*	2005	Diagnósticos mais freqüentes em serviço de emergência para adulto de um hospital universitário	Rev Ciênc Méd	Brazil
Azhar AA *et al*	2000	Patient attendance at a major accident and emergency department: Are public emergency services being abused?	Med J Malaysia	Malaysia
Bains HS *et al*	2012	A simple clinical score “TOPRS” to predict outcome in pediatric emergency department in a teaching hospital in India	Iran J Pediatr	India
Bamgboye EA *et al*	1990	Mortality pattern at a children’s emergency ward, University College Hospital, Ibadan, Nigeria	Afr J Med Med Sci	Nigeria
Basnet B *et al*	2012	Initial resuscitation for Australasian Triage Scale 2 patients in a Nepalese emergency department	Emerg Med Australas	Nepal
Batistela S *et al*	2008	Os motivos de procura pelo Pronto Socorro Pediátrico de um Hospital Universitário referidos pelos pais ou responsáveis	Semina: Ciênc Biológicas Saúde	Brazil
Bazaraa HM *et al*	2012	Profile of patients visiting the pediatric emergency service in an Egyptian university hospital	Pediatr Emerg Care	Egypt
Ben Gobrane HLB *et al*	2012	Motifs du recours aux services d’urgence des principaux hôpitaux du Grand Tunis	East Mediterr Health J	Tunisia
Berraho M *et al*	2012	Les consultations non approprieés aux services des urgences: étude dans un hôpital provincial au Maroc	Prat Organ Soins	Morocco
Boff JM *et al*	2002	Perfil do Usuário do Setor de Emergência do Hospital Universitário da UFSC	Rev Contexto Saude	Brazil
Boros MJ	2003	Emergency medical services in St. Vincent and the Grenadines	Prehosp Emerg Care	St. Vincent and the Grenadines
Bresnahan KA *et al*	1995	Emergency medical care in Turkey: current status and future directions	Ann Emerg Med	Turkey
Brito MVH *et al*	1998	Pronto-atendimento de adultos em serviço de saúde universitário: um estudo de avaliação	Rev Adm Publica	Brazil
Brito MVH *et al*	2012	Perfil da demanda do serviço de urgência e emergência do hospital pronto socorro municipal- Mario Pinotti	Rev Paraense Med	Brazil
Brown MD	1999	Emergency medicine in Eritrea: rebuilding after a 30-year war	Am J Emerg Med	Eritrea
Bruijns S *et al*	2008	A prospective evaluation of the Cape triage score in the emergency department of an urban public hospital in South Africa	Emerg Med J	South Africa
Burch V *et al*	2008	Modified early warning score predicts the need for hospital admission and inhospital mortality	Emerg Med J	South Africa
Buys H *et al*	2013	An adapted triage tool (ETAT) at Red Cross War Memorial Children’s Hospital Medical Emergency Unit, Cape Town: an evaluation	S Afr Med J	South Africa
Cander B *et al*	2006	Emergency operation indications in emergency medicine clinic (model of emergency medicine in Turkey)	Adv Ther	Turkey
Carret MLV *et al*	2007	Demand for emergency health service: factors associated with inappropriate use	BMC Health Serv Res	Brazil
Carret MLV *et al*	2011	Características da demanda do serviço de saúde de emergência no Sul do Brasil	Cien Saude Colet	Brazil
Cevik AA *et al*	2001	Update on the development of emergency medicine as a specialty in Turkey	European J Emerg Med	Turkey
Chattoraj A *et al*	2006	A study of sickness & admission pattern of patients attending an emergency department in a tertiary care hospital	J AcadHosp Adm	India
Chukuezi AB *et al*	2010	Pattern of deaths in the adult accident and emergency department of a sub-urban teaching hospital in Nigeria	Asian J Med Sci	Nigeria
Clark M *et al*	2012	Reductions in inpatient mortality following interventions to improve emergency hospital care in Freetown, Sierra Leone	PLoS one	Sierra Leone
Clarke ME	1998	Emergency medicine in the new South Africa	Ann Emerg Med	South Africa
Clem KJ *et al*	1998	United States physician assistance in development of emergency medicine in Hangzhou, China	Ann Emerg Medi	China
Coelho MF *et al*	2010	Analysis of the organizational aspects of a clinical emergency department: a study in a general hospital in Ribeirao Preto, SP, Brazil	Rev Lat Am Enfermagem	Brazil
Coelho MF *et al*	2013	Urgências clínicas: perfil de atendimentos hospitalares	Rev Lat Am Enfermagem	Brazil
Cox M *et al*	2007	Emergency medicine in a developing country: experience from Kilimanjaro Christian Medial Centre, Tanzania, East Africa	Emerg Med Australas	The United Republic of Tanzania
Curry C *et al*	2004	The first year of a formal emergency medicine training programme in Papua New Guinea	Emerg Med Australas	Papua New Guinea
da Silva GS *et al*	2007	Caracterização do perfil da demanda da emergência de clínica médica do hospital universitário da Universidade Federal de Santa Catarina	Arq Catarinenses Med	Brazil
Dalwai M *et al*	2013	Implementation of a triage score system in an emergency room in Timergara, Pakistan	Public Health Action	Pakistan
Damghi N *et al*	2013	Patient satisfaction in a Moroccan emergency department	Intl Arch Med	Morocco
Dan V *et al*	1991	Prise en charge des urgences du nourrisson et de l'enfant: aspects actuels et perspectives d'avenir	Med Afr Noire	Benin
de Souza LM *et al*	2011	Risk classification in an emergency room: agreement level between a Brazilian institutional and the Manchester Protocol	Rev Lat Am Enfermagem	Brazil
De Vos P *et al*	2008	Uses of first line emergency services in Cuba	Health Policy	Cuba
Derlet RW *et al*	2000	Emergency medicine in Belarus	J Emerg Med	Belarus
Dubuc IF *et al*	2006	Adolescentes atendidos num serviço púlico de urgência e emergência: perfil de morbidade e mortalidade	Rev Eletrônica Enfermagem	Brazil
Duru C *et al*	2013	Pattern and outcome of admissions as seen in the paediatric emergency ward of the Niger Delta University Teaching Hospital Bayelsa State, Nigeria	Nigeria J Pediatr	Nigeria
Ekere AU *et al*	2005	Mortality patterns in the accident and emergency department of an urban hospital in Nigeria	Nigeria J Clinic Pract	Nigeria
Enobong EI *et al*	2009	Pattern of paediatric emergencies and outcome as seen in a tertiary hosptial: a five-year review	Sahel Med J	Nigeria
Erickson TB *et al*	1996	Emergency medicine education intervention in Rwanda	Ann Emerg Med	Rwanda
Eroglu SE *et al*	2012	Evaluation of non-urgent visits to a busy urban emergency department	Saudi Med J	Turkey
Fajardo-Oritz G *et al*	2000	Utilización del servicio de urgencias en un hospital de especialidades	Cir Cir	Mexico
Fajolu IB *et al*	2011	Childhood mortality in children emergency centre of the Lagos University Teaching hospital	Nigeria J Pediatr	Nigeria
Fayyaz J *et al*	2013	Missing the boat: odds for the patients who leave ED without being seen	BMC Emerg Med	Pakistan
Furtado BMA *et al*	2004	O perfil da emergência do Hospital da Restauração: uma análise dos possíveis impactos após a municipalização dos serviços de saúde	Revi Bras Epidemiol	Brazil
Gaitan M *et al*	1998	Growing pains: status of emergency medicine in Nicaragua	Ann Emerg Med	Nicaragua
Garg M *et al*	2013	Study of the relation of clinical and demographic factors with morbidity in a tertiary care teaching hospital in India	Int J Crit Illn Inj Sci	India
George IO *et al*	2010	An audit of cases admitted in the children emergency ward in a Nigerian tertiary hospital	Pakistan J Med Sci	Nigeria
Gim U *et al*	2012	Pattern and outcome of presentation at the children emergency unit of a tertiary institution in the Niger Delta region of Nigeria: a one year prospective study	J Med	Nigeria
Goh AY *et al*	2003	Paediatric utilization of a general emergency department in a developing country	Acta Paediatr	Malaysia
Gomide MFS *et al*	2012	Perfil de usuários em um serviço de pronto atendimento	Med (Ribeirão Preto)	Brazil
Gueguen G	1995	Senegal: mise en place d’un mirco ordinateur dans le service d’accueil des urgencies de l’hopital regional de Ziguinchor	Med Trop	Senegal
Halasa W	2013	Family medicine in the emergency department, Jordan	Br J Gen Pract	Jordan
Hanewinckel R *et al*	2010	Emergency medicine in Paarl, South Africa: a cross-sectional descriptive study	Int J Emerg Med	South Africa
Hexom B *et al*	2012	A model for emergency medicine education in post-conflict Liberia	Afr J Emerg Medi	Liberia
Hodkinson PW *et al*	2009	Cross-sectional suvey of patients presenting to a South African urban emergency centre	Emerg Med J	South Africa
House DR *et al*	2013	Descriptive study of an emergency centre in Western Kenya: challenges and opportunities	Afr J Emerg Med	Kenya
Huo X	1994	Emergency care in China	Accid Emerg Nur	China
Ibeziako SN *et al*	2002	Pattern and outcome of admissions in the children's emergency room of the University of Nigeria Teaching Hospital, Enugu	Nigeria J Paediatr	Nigeria
Jacobs PC *et al*	2005	Estudo exploratório dos atendimentos em unidade de emergência em Salvador-Bahia	Rev Assoc Méd Bras	Brazil
Jafari-Rouhi AH *et al*	2013	The Emergency Severity Index, version 4, for pediatric triage: a reliability study in Tabriz Children’s Hospital, Tabriz, Iran	Int J Emerg Med	Islamic Republic of Iran
Jalili M *et al*	2013	Emergency department nonurgent visits in Iran: prevalence and associated factors	Am J Manag Care	Islamic Republic of Iran
Jerius M *et al*	2010	Inappropriate utilization of emergency medical services at Prince Ali Military Hospital	J R Med Serv	Jordan
Ka Sall B *et al*	2002	Les urgences dans un centre hospitalier et universitaire en milieu tropical	Méd Trop	Senegal
Karabocuoglu M *et al*	1995	Analysis of patients admitted to the emergency unit of a university children's hosptial in Turkey	Turk J Pediatr	Turkey
Karim MZ *et al*	2009	A retrospective study of illness and admission pattern of emergency patients utilizing a corporate hospital in Dhaka, Bangladesh: 2006 −2008	ORION Med J	Bangladesh
Khan NU *et al*	2011	Unplanned return visit to emergency department: a descriptive study from a tertiary care hospital in a low-income country	European J Emerg Med	Pakistan
Khan NU *et al*	2007	Emergency department deaths despite active management: experience from a tertiary care centre in a low-income country	Emerg Med Australas	Pakistan
Khan AN *et al*	2003	International pediatric emergency care: establishment of a new specialty in a developing country	Pediatr Emerg Care	Serbia
Kriengsoontornkij W *et al*	2010	Accuracy of pediatric triage at Siriraj Hospital, Bangkok, Thailand	J Medl Ass Thailand	Thailand
Krym VF *et al*	2009	International partnerships to foster growth in emergency medicine in Romania	CJEM	Romania
Lammers W *et al*	2011	Demographic analysis of emergency department patients at the Ruijin Hospital, Shanghai	Emerg Med Int	China
Lasseter JA *et al*	1997	Emergency medicine in Bosnia and Herzegovina	Ann Emerg Med	Bosnia and Herzegovina
Lee S	2011	Disaster management and emergency medicine in Malaysia	West J Emerg Med	Malaysia
Leite JA *et al*	2010	Internaçöes e serviços de urgência e emergência pactuados em hospital geral púlico da Bahia, Brazil	Rev Baiana Saude Publica	Brazil
Lima SBS *et al*	2013	Perfil clínico-epidemiológico dos pacientes internados no prontosocorro de um hospital universitário	Saude (Santa Maria)	Brazil
Lin JY *et al*	2013	Breadth of emergency medical training in Pakistan	Prehosp Disaster Med	Pakistan
Little RM *et al*	2013	Acute care in Tanzania: epidemiology of acute care in a small community medical centre	Afr J Emerg Med	The United Republic of Tanzania
Liu Y *et al*	1994	A preliminary epidemiological study of the patient population visiting an urban ED in the Republic of China	Am J Emerg Med	China
Lopes SLB *et al*	2007	The implementation of the Medical Regulation Office and Mobile Emergency Attendance System and its impact on the gravity profile of non-traumatic afflictions treated in a University Hospital: a research study	BMC Health Serv Res	Brazil
Loria-Castellanos J *et al*	2006	Reanimation unit experience of a second- level hospital in Mexico City	Prehosp Disaster Med	Mexico
Loria-Castellanos J *et al*	2010	Frecuencia y factores asociados con el uso inadecuado de la consulta de urgencias de un hospital	Cir Cir	Mexico
Mabiala-Babela JR *et al*	2007	Consultations et réadmissions avant l’âge d’un mois aux urgences pédiatriques, Brazzaville (Congo)	Arch Pediatr	Congo
Mabiala-Babela JR *et al*	2009	Consultations de nuit aux urgences pédiatriques du CHU de Brazzaville, Congo	Méd Trop	Congo
Mahajan V *et al*	2013	Unexpected hospitalisations at a 23-Hour observation unit in a paediatric emergency department of northern India	J Clin Diagn Res	India
Maharjan RK	2011	Mortality pattern in the emergency department in Tribhuvan University Teaching Hospital, Kathmandu, Nepal	Prehosp Disaster Med	Nepal
Malhotra S *et al*	1992	A study of the workload of the casualty department of a large city hospital	Health Popul Perspect Issues	India
Martinez RQ *et al*	2008	Padecimientos más frecuentemente atendidos en el Servicio de Urgencias Pediátricas en un hospital de tercer nivel	Rev Fac Med Univ Auton Mex	Mexico
Matoussi N *et al*	2007	Profil epidemiologique et prise en charge des consultants des urgences medicales pediatriques de l’hopital d’enfants de Tunis	Tunis Med	Tunisia
Maulen-Radovan I *et al*	1996	PRISM score evaluation to predict outcome in paediatric patients on admission at an emergency department	Arch Med Res	Mexico
Mbutiwi Ikwa Ndol F *et al*	2013	Facteurs pre ´dictifs de la mortalite ´ des patients admis aux urgences me ´dicales des cliniques universitaires de Kinshasa	Rev Epidemiol Sante Publique	Democratic Republic of the Congo
McDonald A *et al*	2005	Emergency medicine in Jamaica	CJEM	Jamaica
McDonald A *et al*	2000	Morbidity pattern of emergency room patients in Jamaica	West Indian Med J	Jamaica
Medina J *et al*	2007	Triage: experiencia en un Servicio de Urgencias Pediátricas	Rev Soc Boliv Pediatr	Paraguay
Meijas CLA	2011	La atención de urgencia y la dispensarización en el Policlínico Universitario Docente de Playa	Rev Cubana Med Gen Integral	Cuba
Melo EMC *et al*	2007	O trabalho dos pediatras em um serviço público de urgências: fatores intervenientes no atendimento	Cad Saude Publica	Brazil
Meskin S *et al*	1997	Delivery of emergency medical services in Kathmandu, Nepal	Ann Emerg Med	Nepal
Mijinyawa MS	2010	Pattern of medical emergency utilisation in a Nigeria tertiary health institution: a preliminary report	Sahel Med J	Nigeria
Miranda NA *et al*	2012	Caracterização de crianças atendidas no pronto-socorro de um hospital universitário	Rev Eletrônica Gestão Saúde	Brazil
Molyneux E *et al*	2006	Improved triage and emergency care for children reduces inpatient mortality in a resource-constrained setting	Bull World Health Organ	Malawi
Morton TD	1992	A perspective on emergency medicine in the developing world	J Emerg Med	Malaysia
Mullan PC *et al*	2013	Reduced overtriage and undertriage with a new triage system in an urban accident and emergency department in Botswana: a cohort study	Emerg Med J	Botswana
Muluneh D *et al*	2007	Analysis of admissions to the pediatric emergency ward of Tikur Anbessa Hospital in Addis Ababa, Ethiopia	Ethiop J Health Dev	Ethiopia
Musharafieh R *et al*	1996	Development of emergency medicine in Lebanon	Ann Emerg Med	Lebanon
Naidoo DK *et al*	2014	An evaluation of the Triage Early Warning Score in an urban accident and emergency department in KwaZulu-Natal	S Afr Family Pract	South Africa
Nelson BD *et al*	2005	Integrating quantitative and qualitative methodologies for the assessment of health care systems: emergency medicine in post-conflict Serbia	BMC Health Serv Res	Serbia
Nemeth J *et al*	2001	Emergency medicine in Eastern Europe: the Hungarian experience	Ann Emerg Med	Hungary
Nkombua L	2008	The practice of medicine at a district hospital emergency room: Middelburg Hospital, Mpumalanga Province	S Afr Family Pract	South Africa
O'Reilly G *et al*	2010	The dawn of emergency medicine in Vietnam	Emerg Med Australas	Viet Nam
Ogah OS *et al*	2012	A two-year review of medical admissions at the emergency unit of a Nigerian tertiary health facility	Afr J Biomed Res	Nigeria
Ogunmola OO *et al*	2013	Mortality pattern in adult accident and emergency department of a tertiary health centre situated in a rural area of developing country	J Dent Med Sci	Nigeria
Ohara K *et al*	1994	Investigación sobre pacientes atendidos en el servicio de emergencia del Hospital Escuela	Rev Med Hondur	Honduras
Okolo SN *et al*	2002	Health service auditing and intervention in an emergency paediatric unit	Nigeria J Paediatr	Nigeria
Oktay C *et al*	2003	Appropriateness of emergency department visits in a Turkish university hospital	Croat Med J	Turkey
Olivati FN *et al*	2010	Perfil da demanda de um pronto-socorro em um município do interior do estado de São Paulo	Rev Fac Odontol Univ Passo Fundo	Brazil
Oliveira GN *et al*	2011	Profile of the population cared for in a referral emergency unit	Rev Lat Am Enfermagem	Brazil
Onwuchekwa AC *et al*	2008	Medical mortality in the accident and emergency unit of the University of Port Harcourt Teaching Hospital	Nigeria J Med	Nigeria
Orimadegun AE *et al*	2008	Comparison of neonates born outside and inside hospitals in a children emergency unit, southwest of Nigeria	Pediatr Emerg Care	Nigeria
Osuigwe AN *et al*	2002	Mortalty in the accident and emergency unit of Nnamdi Azikiwe University Teaching Hospital, NNEWI: patterns and factors involved	Nigeria J Clin Pract	Nigeria
Parekh KP *et al*	2013	Who leaves the emergency department without being seen? A public hospital experience in Georgetown, Guyana	BMC Emerg Med	Guyana
Partridge RA	1998	Emergency medicine in West Kazakhstan, CIS	Ann Emerg Med	Kazakhstan
Patil A	2013	Analysis of pattern of emergency cases in the casualty of a university	Indian J Forensic Med Toxicol	India
Peixoto BV *et al*	2013	The harsh reality of children and youth emergency care showing the health status of a city	Rev Paul Pediatr	Brazil
Pekdemir M *et al*	2010	No significant alteration in admissions to emergency departments during Ramadan	J Emerg Med	Turkey
Periyanayagam U *et al*	2012	Acute care needs in a rural Sub-Saharan African emergency centre: a retrospective analysis	Afr J Emerg Med	Uganda
Pinilla MMA *et al*	2009	Demandas inadecuadas en urgencias e identificación del uso inapropriado de la hospitalización en el Centro Piloto de ASSBASALUD ESE. en Manizales. año 2008	Arch Med	Colombia
PoSaw LL *et al*	1998	Emergency medicine in the new Delhi Area, India	Ann Emerg Med	India
Raftery KA	1996	Emergency medicine in southern Pakistan	Ann Emerg Med	Pakistan
Ramalanjaona G	1998	Emergency medicine in Madagascar	Ann Emerg Med	Madagascar
Rashidi A *et al*	2009	Demographic and clinical characteristics of red tag patients and their one-week mortality rate from the emergency department of the Hospital Universiti Sains Malaysia	South-east Asian J Trop Med Public Health	Malaysia
Rehmani R	2004	Emergency section and overcrowding in a university hospital of Karachi, Pakistan	J Pak Med Assoc	Pakistan
Reynolds TA *et al*	2012	Most frequent adult and pediatric diagnoses among 60 000 patients seen in a new urban emergency department in Dar es Salaam, Tanzania	Ann Emerg Med	The United Republic of Tanzania
Reynolds TA *et al*	2012	Emergency care capacity in Africa: a clinical and educational initiative in Tanzania	J Public Health Policy	The United Republic of Tanzania
Ribeiro RCH *et al*	2013	Stay and outcome of the clinical and surgical patient in the emergency service	Rev Enfermagem	Brazil
Riccetto AGL *et al*	2007	Sala de emergência em pediatria: casuística de um hospital universitário	Revi Paul Pediatr	Brazil
Richards JR	1997	Emergency medicine in Vietnam	Ann Emerg Med	Viet Nam
Robison JA *et al*	2012	Decreased pediatric hospital mortality after an intervention to improve emergency care in Lilongwe, Malawi	Pediatr	Malawi
Rodriguez JCM *et al*	2012	Aplicación de los criterios de ingreso a la unidad de reanimación en el servicio de urgencias de adultos del Hospital General «La Raza»	Arch Medi Urg Méx	Mexico
Rodriguez JP *et al*	2001	“Filtro Sanitario” en las urgencias médicas. Un problema a reajustar	Rev Cubana Med	Cuba
Rosa TP *et al*	2011	Perfil dos pacientes atendidos na sala de emergência do pronto Socorro de um hospital universitário	Rev Enfermagem UFSM	Brazil
Rosedale K *et al*	2011	The effectiveness of the South African Triage Score (SATS) in a rural emergency department	S Afr Med J	South Africa
Rytter MJH *et al*	2006	Effects of armed conflict on access to emergency health care in Palestinian West Bank: systematic collection of data in emergency departments	BMJ	West Bank and Gaza Strip
Salaria M *et al*	2003	Profile of patients attending pediatric emergency service at Chandigarh	Indian J Pediatr	India
Salgado RMP *et al*	2010	Perfil dos pacientes pediátricos atendidos na emergência de um hospital universitário	Pediatr (Sao Paulo)	Brazil
Santhanam I *et al*	2002	Mortality after admission in the pediatric emergency department: a prospective study from a referral children’s hospital in southern India	Pediatr Critical Care Med	India
Savitsky E *et al*	2000	Emergency medical services development in the Seychelles Islands	Am J Emerg Med	Seychelles
Selasawati HG *et al*	2004	Inappropriate utilization of emergency department services in Universiti Sains Malaysia Hospital	Med J Malaysia	Malaysia
Sentilhes-Monkam A	2011	Les services d’accueil des urgences ont-ils un avenir en Afrique de l’ouest? Exemple à l’hôpital principal de Dakar	Sante Publique	Senegal
Shakhatreh H *et al*	2009	Patient satisfaction in emergency department at King Hussein Medical Center	J R Med Serv	Jordan
Shakhatreh H *et al*	2003	Use and misuse of accident and emergency services at Queen Alia Military Hospital	J R Med Serv	Jordan
Simons DA *et al*	2010	Adequação da demanda de crianças e adolescentes atendidos na Unidade de Emergência em Maceió, Alagoas, Brasil	Rev Bras Saúde Matern Infant	Brazil
Singhi S *et al*	2004	Comparison of pediatric emergency patients in a tertiary care hospital vs a community hospital	Indian Pediatr	India
Singhi S *et al*	2003	Pediatric emergencies at a tertiary care hospital in India	J Trop Pediatr	India
Smadi BY *et al*	2005	Inappropriate use of emergency department at Prince Zeid Ben Al-Hussein Hospital	J R Med Serv	Jordan
Soleimanpour H *et al*	2011	Emergency department patient satisfaction survey in Imam Reza Hospital, Tabriz, Iran	Int J Emerg Med	Islamic Republic of Iran
Souza BC *et al*	2009	Perfil da Demanda do Departamento de Emergência do Hospital Nossa Senhora da Conceição – Tubarão – SC	Arq Catarinenses Med	Brazil
Tannebaum RD *et al*	2001	Emergency Medicine in Southern Brazil	Ann Emerg Med	Brazil
Taye BW *et al*	2014	Quality of emergency medical care in Gondar University Referral Hospital, north-west Ethiopia: a survey of patient's perspectives: a survey of patients’ perspectives	BMC Emerg Med	Ethiopia
Tiemeier K *et al*	2013	The effect of geography and demography on outcomes of emergency department patients in rural Uganda	Ann Emerg Med	Uganda
Tinaude O *et al*	2010	Health-care-seeking behaviour for childhood illnesses in a resource-poor setting	J Paediatr Child Health	Nigeria
Tintinalli J *et al*	1998	Emergency care in Namibia	Ann Emerg Med	Namibia
Topacoglu H *et al*	2004	Analysis of factors affecting satisfaction in the emergency department: a survey of 1 019 patients	Adv Ther	Turkey
Traoré A *et al*	2002	Les urgences médicales au Centre hospitalier national Yalgado Ouédraogo de Ouagadougou : profil et prise en charge des patients	Cah Etud Rech Francophones / Santé	Burkina Faso
Trejo JA *et al*	1999	El servicio de urgencias en un hospital de tercer nivel. Su comportamiento durante cinco años: estudio preliminar	Med Interna Mex	Mexico
Tsiperau J *et al*	2010	The management of paediatric patients in a general emergency department in Papua New Guinea	P N G Med J	Papua New Guinea
Ugare GU *et al*	2012	Epidemiology of death in the emergency department of a tertiary health centre south-south of Nigeria	Afr Health Sc	Nigeria
Veras JEG *et al*	2011	Profile of children attended in emergency according to the risk classification: a documental study	Online Bras J Nurs	Brazil
Veras JEG *et al*	2009	Analise das causas de atendimento de crianças e adolescentes menores de 15 anos em pronto-atendimento de um hospital secundário de Fortaleza	Congr Bras Enfermagem	Brazil
Wang L *et al*	2011	Application of emergency severity index in pediatric emergency department	World J Emerg Med	China
Webb HR *et al*	2001	Emergency medicine in Ecuador	Am J Emerg Med	Ecuador
Williams EW *et al*	2008	The evolution of emergency medicine in Jamaica	West Indian Med J	Jamaica
Wright SW *et al*	2000	Emergency medicine in Ukraine: challenges in the post-Soviet era	Am J Emerg Med	Ukraine
Yaffee AQ *et al*	2012	Bypassing proximal health care facilities for acute care: a survey of patients in a Ghanaian accident and emergency centre	Trop Med Int Health	Ghana
Yildirim C *et al*	2005	Patient satisfaction in a university hospital emergency department in Turkey	Acta Med	Turkey
Zhou JC *et al*	2012	High hospital occupancy is associated with increased risk for patients boarding in the emergency department	Am J Med	China

Notes: Further information about the studies can be obtained from corresponding author. Additional data were obtained through personal communications from: Botswana (1), Cameroon (1), Ghana (1), Lebanon (1), Liberia (1), Madagascar (1) and South Africa (10).

Table 2 presents the key metrics for the facilities. Median mortality within the emergency departments – of the 65 facilities that reported the relevant data – was 1.8% overall and higher in the 19 paediatric facilities (4.8%) than in the 46 adult or general facilities (0.7%). Across World Bank regions that we investigated, mortality was highest in sub-Saharan Africa (3.4%; IQR: 0.5–6.3%; *n* = 44), especially in east, central or west Africa (4.8%; IQR: 3.3–8.4%; *n* = 30). Paediatric facilities in sub-Saharan Africa had a median mortality of 5.1% (IQR: 3.5–11.1%; *n* = 15). Mortality in emergency facilities was also high in Latin America. Two facilities in Brazil were major contributors to this high rate, with mortality of 7.4%^32^ and 3.9%.^33^ These centres also reported long inpatient stays: one facility reported a median length of stay of three days,^32^ whereas the other reported that 21% of patients stayed in the emergency department for more than five days.^33^ Lengths of stay were only reported for 15 facilities and for these, the median value was 7.7 hours (IQR: 3.3–40.8). As mortality data were only available for nine of these 15 facilities, it was not possible to formally investigate the relationship between length of stay and mortality. The five sub-Saharan African facilities that recorded length of stay reported a median stay of 17 hours (IQR: 16.9–18.0). Additional data comparing mortality, patient volumes and admission are available from the corresponding author.

**Table 2:** Key quantitative data for emergency departments, 59 low- and-middle-income countries, 1990–2014

Metric	Facility type	Units^a^	All regions	Sub-Saharan Africa	South Asia, East Asia & Pacific	Middle East & North Africa	Latin America & Caribbean	Europe & Central Asia
No. of beds	All	*n*	60	24	20	4	9	3
	All	Median (IQR)	14 (8–22)	9 (8–14)	21 (15–23)	11 (8–25)	17 (12–22)	16 (16–27)
Annual patient volume (thousands)	All	*n*	173	64	35	24	42	8
	All	Median (IQR)	30.0 (10.3–60.0)	13.6 (3.4–29.8)	36.5 (8.3–70.0)	49.0 (34.0–68.8)	52.4 (26.0–87.0)	33.8 (15.3–72.1)
	General and adult	Median (IQR)	36.9 (15.8–64.2)	16.7 (5.1–35.3)	50.0 (29.2–81.2)	53.6 (34.0–79.0)	59.7 (31.0–89.1)	36.5 (14.6–82.1)
	Paediatric	Median (IQR)	7.2 (2.3–31.6)	3.1 (2.0–7.5)	5.6 (2.2–7.6)	43.0 (22.1–44.3)	27.5 (13.5–68.1)	31.0 (NA)
Admission, %	All	*n*	78	26	16	15	20	1
	All	Median (IQR)	20.0 (10.1–42.8)	24.5 (16.5–46.9)	26.0 (15.0–38.7)	18.2 (10.1–22.2)	11.1 (3.9–20.7)	50.0 (NA)
	General and adult	Median (IQR)	18.8 (9.4–40.1)	24.5 (15.8–46.5)	24.2 (15.0–36.3)	14.9 (7.7–18.9)	10.2 (3.9–19.5)	50.0 (NA)
	Paediatric	Median (IQR)	22.2 (10.7–44.3)	33.2 (20.6–65.2)	32.5 (14.0–43.0)	21.8 (15.7–28.6)	14.3 (6.4–35.4)	NA (NA)
Mortality,%^b^	All	*n*	65	44	9	5	7	NA
	All	Median (IQR)	1.8 (0.2–5.1)	3.4 (0.5–6.3)	0.3 (0.2–0.8)	0.7 (0.2–2.1)	2.0 (0.1–7.4)	NA (NA)
	General and adult	Median (IQR	0.7 (0.2–3.9)	0.9 (0.2–4.8)	0.3 (0.2–0.5)	0.5 (0.2–1.4)	2.0 (0.1–7.4)	NA (NA)
	Paediatric	Median (IQR)	4.8 (2.3–8.4)	5.1 (3.5–11.1)	0.8 (< 0.1–2.7)	7.8 (NA)	NA (NA)	NA (NA)

IQR: interquartile range; NA: not available.

^a^ For each metric and region, the number of facilities for which the relevant data were available (*n*) is indicated.

^b^ Within the emergency department.

Data sources listed in Table 1, (available at: http://www.who.int/bulletin/volumes/93/14/07-148338).

Median annual patient volume was 30 021 (IQR: 10 296–60 000) among 173 facilities reporting these data. Volume was lower in the nine rural facilities (16 468; IQR: 3429–44 395) than in the 164 urban ones (31 000; IQR: 10 994–61 313). The 17 paediatric facilities in Sub-Saharan Africa had relatively low patient volumes with a median annual patient volume of 3129 (IQR: 2009–7479). The median inpatient admission was 20% (IQR: 10–43%; *n* = 78) and the median number of beds in the emergency department was 14 (IQR: 8–22; *n* = 60). The median age of patients attending non-paediatric facilities was 35 years (IQR: 6.9–41.0; *n* = 51) and a median of 55.7% (IQR: 50.0–59.2%; *n* = 93) were male. The corresponding values for paediatric facilities were 3.2 years (IQR: 2.8–3.4; *n* = 13) and 58.3% (IQR: 55.4–60.1%; *n* = 27), respectively.

Table 3 summarizes the training of providers staffing the 102 facilities for which provider data were available. Care in 67 (66%) of these facilities was provided either by trainees or by physicians whose level of training was not specified. In only 29 (28%) of facilities were attending or consultant-level physicians available full-time; in 19 other facilities, physicians were only available in daytime hours. Eighteen facilities were staffed by specialty-trained emergency physicians, but in only four facilities were emergency physicians available at all times – one in the United Republic of Tanzania (unpublished observations, 2014), one in Pakistan^34^ and two in Nicaragua.^35^ One facility provided specialized emergency training to non-physician providers staffing the emergency department.^36^ In another facility, medical students practising alone were primarily responsible for providing emergency care during most of the day.^37^ Patients had to navigate through a wide range of options to obtain emergency care and financial factors played a major role in determining what kind of care they received (details available from the corresponding author).

**Table 3:** Training of providers of emergency care included in systematic analysis, 49 low- and-middle-income countries, 1991–2014

Region,^a^ country	No. of facilities			
	Non-physician or medical student	Physician in training or with unspecified level of training	Attending physician or consultant	Emergency physician
**Sub-Saharan Africa**				
Botswana	–	1	–	1^b^
Burkina Faso	–	1	–	–
Cameroon	–	1	1^b^	–
Congo	1	–	1^b^	–
Eritrea	–	1	–	–
Ghana	–	2	–	–
Kenya	–	1	–	–
Liberia	–	1	1^b^	–
Madagascar	–	1	1	–
Malawi	–	–	2	–
Namibia	–	1	–	–
Nigeria	–	4	2	1^b^
Rwanda	–	1	–	–
Seychelles	–	1	–	–
Sierra Leone	–	1	–	–
South Africa	–	8	4	1^b^
Sudan	–	2	–	2^c^
Uganda	1^b^	–	–	–
United Republic of Tanzania	–	2	–	1
**South Asia, East Asia & Pacific**				
China	–	2	2^b^	–
India	–	3	7^c^	–
Kazakhstan	–	1	–	–
Malaysia	–	2	–	1^b^
Nepal	–	4	3^d^	1^b^
Pakistan	–	2	1	1
Papua New Guinea	–	1	–	–
Viet Nam	–	2	–	–
**Latin America & Caribbean**				
Brazil	–	1	3	–
Cuba	–	–	2	–
Ecuador	–	3	1^b^	–
Guyana	–	1	–	–
Jamaica	–	–	1	1^b^
Mexico	–	1	–	1^b^
Nicaragua	–	–	–	2
Paraguay	–	1	–	1
Saint Vincent and the Grenadines	–	1	–	–
**Middle East & North Africa**				
Egypt	–	1	1^b^	–
Islamic Republic of Iran	–	1	–	–
Jordan	–	4	3^d^	1^b^
Lebanon	–	–	1	1^b^
Morocco	–	1	1^b^	–
Tunisia	–	1	1^b^	–
**Europe & Central Asia**				
Belarus	–	1	1^b^	–
Bosnia and Herzegovina	–	2	–	1^b^
Hungary	–	–	1	–
Romania	–	–	1	1^b^
Serbia	–	–	1	–
Turkey	–	1	2^b^	1^b^
Ukraine	–	1	3^b^	–

^a^ Regions according to the World Bank.^30^

^b^ Provider only available part-time in the facility or one of the facilities.

^c^ Provider only available part-time in two of the facilities.

^d^ Provider only available part-time in three of the facilities.

Data sources listed in Table 1, (available at: http://www.who.int/bulletin/volumes/93/14/07-148338).

## Discussion

While only a small set of metrics on the delivery of emergency care were reported consistently across facilities, we were able to draw some conclusions on the state of emergency care in low-resource settings.

First, large numbers of patients presented to health facilities seeking emergency care. While there was a wide range in annual patient volumes – from just 451 in a paediatric emergency department in Nigeria^38^ to 273 182 in a general emergency department in Turkey^39^ – they were approximately 10 times higher than the corresponding caseloads observed in primary care settings in sub-Saharan Africa and Asia.^9^

Second, patients seeking emergency care were generally young and free of chronic conditions. This is in contrast to the growing burden of elderly patients with multiple chronic conditions seen in the emergency departments of high-income countries.^40^ Therefore, interventions to decrease mortality and morbidity in emergency settings of LMICs could dramatically increase life-years saved and productivity.

Third, the mortality recorded in emergency departments in LMICs was many times higher than generally reported in high-income countries.^40^^–^^42^ A recent report on emergency departments in the USA documented a mean mortality within the departments of 0.04%.^40^

Fourth, most providers of emergency care in LMICs had no specialty training in emergency care. This observation was expected given the general shortages in human resources for health in most of these countries.^43^ Such shortages may be particularly pronounced in emergency settings, where the work is demanding and salaries are often poor. Most governments do not include emergency medicine in their medical education priorities.

What implications do these results have for LMICs? We made a rough calculation for Nigeria, where we identified relevant studies in 21 facilities and mean annual patient volume of 3000 and 5–7% mortality. If we assume that the approximately 1000 teaching and general hospitals^44^ in the country have the same mean annual patient volume and mortality, then out of the 1.6 million deaths recorded annually in Nigeria^45^ an estimated 10–15% occur in emergency departments. This estimate – and the observation that most emergency departments in LMICs are run by providers with no speciality training in emergency care – illustrates the opportunity to improve emergency care in LMICs. It is likely that relatively simple interventions to facilitate triage and improve patient flow, communication and the supervision of junior providers (Box 1) could lead to reductions in the mortality associated with emergency care.^46^^–^^49^

Box 1 **Interventions to reduce mortality from medical emergencies in four low- or middle-income countries**

**Rural districts in Cambodia and northern Iraq**

Local paramedics and lay first responders were trained to provide field care for trauma. After the intervention, the trauma mortality decreased from 40% to 15%.^46^

**Queen Elizabeth Hospital, Malawi**

The paediatric clinic was physically restructured to streamline operations, clinical staff were trained in emergency care and triage and cooperation between the inpatient and outpatient services was improved. After the intervention, mortality within 24 hours of presentation decreased from 36% to 13%.^47^

**Ola During Children’s Hospital, Sierra Leone**

A triage unit was established in the outpatient department and the emergency and intensive care units were combined. Clinical staff were trained in emergency care and triage, with experienced nursing and medical officers required to be present at all times. Equipment and record keeping were also enhanced. After the intervention, inpatient mortality decreased from 12% to 6%.^48^

**Kamuzu Central Hospital, Malawi**

The paediatric clinic allocated senior medical staff to supervise emergency care and implemented formal triage procedures, with an emphasis on early patient treatment and stabilization before transfer to the inpatient ward. Inpatient mortality within two days of admission decreased, from 5% to 4%.^49^

Our data illustrate the unique cost–benefit profile of investments in emergency care. Although disease and injury prevention are key functions of all health systems, acute health problems – e.g. myocardial infarction, sepsis and trauma – continue to occur in all countries. With the same amount of resources, it is likely that more lives could be saved in a paediatric emergency facility with mortality between 12% and 21%^50^^–^^52^ than in paediatric primary-care clinics in similar settings – which generally see just a few critically-ill children per clinic per week (unpublished observations, 2015). There is thus a clear case for investing in emergency care in LMICs, to complement existing efforts to strengthen primary and preventive care.

### Implications for policy

What is needed to strengthen emergency care in LMICs? First, a better understanding of the conditions that drive patients to seek such care is crucial. We documented high patient volumes and mortality but did not identify the diseases or the conditions that drive these metrics. While useful estimates of the burden of acute conditions may be produced in mathematical models,^53^ the setting of specific clinical and policy priorities remains difficult because of the scarcity of relevant data.

Second, once we have a better understanding of the burden of acute disease, interventions known to be effective in high-income settings – e.g. trauma resuscitation training – must be adapted to LMICs and critically assessed. Some effective interventions to decrease mortality in emergencies (Box 1) may only require the improved use of existing system components, with the minimal input of new material resources. However, assessing the effectiveness of such interventions by rigorous experimental or quasi-experimental methods requires additional funding. Although before-and-after comparisons may be easier, they are also vulnerable to a range of biases.^54^

Third, international organizations must accelerate efforts to develop consensus on the essential components of systems for emergency care. Policy-makers who wish to assess their emergency systems and set priorities for development need technical guidance. WHO’s framework on systems of trauma care is one useful model for this broad agenda.^55^

Finally, improvements to emergency care in LMICs will require advances in data collection. The development of a minimum set of indicators for emergency care in LMICs would facilitate research and quality improvement.^21^ Several actors are improving platforms for data collection in LMICs. For example, the African Federation for Emergency Medicine is building consensus around a medical chart that has been purpose-designed to capture data for clinicians, administrators and researchers in LMICs. A novel data collection platform has been implemented for trauma care in a large teaching hospital in the United Republic of Tanzania, with promising early results. The systematic integration of routine data collection into care delivery settings should help ensure that interventions are – and remain – effective.

### Limitations

The most important limitation of our study is the general paucity of data on emergency care. After screening over 40 000 published reports, we identified relevant data from only 192 facilities spread across 59 LMICs. For comparison, there are about 5000 emergency departments in the USA.^56^ The facilities we identified were largely urban and academic – as might be expected given that our search strategy relied mainly on published reports. Broader reporting biases may also have affected our results. For example, facilities with fewer resources may be relatively unlikely to collect and publish data and facilities with exceptionally high levels of mortality may be relatively unlikely to publish those levels. Thus, our results are likely to present an optimistic view of the state of emergency care in LMICs.

Regional comparisons must be viewed with caution, given the geographical variation in facility characteristics and reporting practices. For example, emergency departments in which patients have exceptionally long lengths of stay will probably also have exceptionally high mortality – since patients who stay longer in the department are more likely to die in the department. Although a lack of relevant data prevented us from investigating this relationship, the median length of stay in our sample – albeit in the small number of facilities that reported lengths of stay – was only 7.7 hours. It therefore seems unlikely that prolonged stays alone could have accounted for the high levels of mortality that we observed.

Other limitations were our search strategy, which relied on the presence of at least one word that began with “emerg” in the title, keywords or abstract of an article. While this made a difficult search problem tractable, it may also have excluded some relevant studies. Also, lack of data standardization across facilities and countries probably biased our results. For example, standardized measures of mortality – e.g. the percentage of patients that died with 24 hours of their presentation – were seldom reported, probably because of the difficulties of following-up patients after they leave the emergency department. The maximum age for a so-called paediatric patient also varied widely across studies, from five to 19 years.^57^^,^^58^

## Conclusion

Emergency facilities in LMICs serve a large, young patient population with high levels of critical illnesses and mortality. This suggests that emergency care should be a global health priority. The cost–benefit ratio for improvements in emergency care is likely to be highly favourable, given the high volume of patients for whom high-quality care could be the difference between life and death. There are likely to be substantial opportunities to improve care and impact outcomes, in ways that could be rigorously evaluated with manageable sample sizes.
